# New Appliances and Things Medical

**Published:** 1901-06-22

**Authors:** 


### NEW APPLIANCES AND THINGS MEDICAL.

[We shall be glad to receive, at our Office, 28 & 29 Southampton Street, Strand, London, W.C., from the manufacturers, specimens of all new preparations and appliances which may be brought out from time to time.]

### SULPHUME.

(A. Boake, Roberts and Co., Limited, Stratford, London, E.)

Sulphume is anhydrous sulphurous acid liquefied by cold and pressure, and supplied in hermetically sealed tins, containing 12 and 20 ozs. of the fluid. It may also be obtained in large quantities in copper cylinders or steel bottles. This method of obtaining the fumes will be appreciated by those who heretofore have employed the clumsy expedient of burning sulphur. The small tins which are designed for domestic uses, such as the disinfection of rooms occupied by fever patients, are employed in the following simple manner: The chamber to be disinfected is sealed up in the usual manner, all but the doorway, by which the operator is to make his exit. Means, however, must be taken for closing up this aperture immediately after his departure. The tin is then taken in the left hand pointing away from the operator and opened by cutting a small lead pipe with a strong knife, the liquid will immediately rush out, and if placed, opening downwards, in an empty washing basin, which must be arranged all ready beforehand, will continue to flow out and evaporate, till all the contents are exhausted. At the end of eight hours the room can be opened, and the process of disinfection is at an end. Not one of the least advantages of this method, as compared with the old one of burning sulphur, is the immunity from risk of fire.

### SCRUBB'S CLOUDY AMMONIA AND ANTISEPTIC SKIN SOAP.

(Scrubb and Co., Guildford Street, Lambeth, S.E.)

Scrubb's cloudy ammonia is not only a most valuable substitute for soda in all washing operations, but it possesses properties which are not possessed by other forms of alkali For removing grease of all kinds some form of alkali is necessary, but ammonia is the only one which, if used in excess, does not leave some sort of solid residue on drying. This is especially important when the object washed happens to be the skin of an individual. Any form of soda, if allowed to dry upon the skin, leaves a solid residue, which must eventually act deleteriously on its condition. Ammonia, on the other hand, immediately evaporates, leaving nothing behind; further than this, it acts as a useful stimulant to skin or hair, and is a most invigorating addition to the bath. We strongly recommend those of our readers who appreciate these advantages to use Scrubb's antiseptic skin soap in conjunction with the cloudy ammonia, both in the bath and for the ordinary ablutions of the toilette.

